# Single neurons needed for brain asymmetry studies

**DOI:** 10.3389/fgene.2013.00311

**Published:** 2014-01-16

**Authors:** Athina Samara

**Affiliations:** Department of Physiology, Institute of Basic Medical Sciences, University of OsloOslo, Norway

**Keywords:** RNA-seq, asymmetry, neurons, proteomics, genomics, heterogeneity, single-neuron diversity

The complexity of the human brain is inconceivable, and can only be conveyed in numbers; for example, the 100 billion neurons comprising it, without taking into account the numerous non-neuronal cells i.e., the glia and the astrocytes. Each neuron may communicate with another 10,000 neurons, with trillions of intercellular junctions (synapses) established by adulthood; synapses demonstrating plasticity and prone to change over time. Human genome sequences foresee that each neuron could express over 20,000 protein coding genes and thousands of non-coding RNAs. Recent advances in high-throughput techniques such as RNA-Seq, proteomics, bioinformatics and meta-analyses of the resulting data, unveil deeper brain heterogeneity than what researchers had suspected.

Brain asymmetry in terms of neuronal heterogeneity, makes the examination of the connections and biochemical steady state of each neuron seemingly insurmountable. The Allen Institute for Brain Science (http://www.alleninstitute.org/) educates naïve minds and experts further: neuronal vs. non-neuronal populations, heterogeneity due to developmentally regulated specific gene expression in different brain regions to attain brain maturation. Scrutinizing the highly polarized nature of neurons being responsible for uneven distribution of the gene products; thus compartments such as soma, axon, dendrites and synapses contain unique pools of RNAs and proteins and may have different signaling networks.

Earlier single neuron transcriptome studies in *Caenorhabditis elegans* demonstrated that even functionally related neurons express >1000 genes in a differential manner (Etchberger et al., 2007). Given that the transcriptome of each single neuron undertakes dynamic changes in response to external and developmental signals, single neuron RNA-Seq can give undiluted cell-specific information, compared to pooling groups of neurons. Single-cell genome analysis is inherently challenging, and RNA-Seq does not depend on genome annotation for prior probe selection; these differences with microarrays lower RNA-Seq bias. Both RNA-Seq and microarrays depend on genome amplification (cDNA) before measurement, hence validation is impossible because the single-cell's genome before amplification cannot be determined [for neuron specific genomics proteomics reviews see Hebenstreit et al. (2012); Kadakkuzha and Puthanveettil (2013)]. According to the work of Qiu et al. (2012), electrophysiology, and single mouse neuron microaspiration gave ~10 pg of RNA both in brain slices or 12DIV neuronal cultures. RNA-Seq confirmed that morphologically identical mouse neurons, even from the same brain region, have distinct expression patterns. Their data suggested that the observed differences among different single neuron analyses of gene expression are mostly due to cell-to-cell phenotypic variability rather than noise due to technical issues. Undoubtedly, the mRNA content residing in distal compartments (e.g., axons, dendrites) might not be captured by microaspiration (Eberwine, 2001; Eberwine et al., 2001; Batish et al., 2012). Qiu et al advised laser capture microdissection as an alternative method to single-cell aspiration for single cell studies.

Single neuron transcriptomics optimization methods are currently under scrutiny. But the full molecular signature of a neuron goes beyond genomics and transcriptomics, as it embraces the proteome. Changes in the proteome are intricate, not only by the decrease or increase in the number of copies of protein molecules of interest, but also by changes in the functional states of the proteins; states such as phosphorylation, glycosylation etc, regulated by their association with other proteins and protein modifications. A single-cell functional proteomics assay measures the quantity of such a functional state of a given protein or panel of proteins and the changes in a cell's activity (such as neuronal migration) or activation/inhibition of protein signaling networks. [A recent review by Wei et al covers emerging technologies and associated applications, designed to characterize cellular heterogeneity by single-cell functional proteomics (Wei et al., 2013)].

The proteome of cells between different brain regions is known to differ greatly, giving rise to a multitude of studies of brain asymmetry. However, unlike detecting low copies of RNAs by linear amplification, there are no amplification methodologies for proteins, thus low abundance proteins may be lost during sample analysis [for methods in neuroproteomics (Craft et al., 2013)].

A series of emerging technologies enabled the examination of changes in proteome at the single-cell level. Studies from the Sweedler group bridging chemistry and neurobiology, have established the use of mass spectrometry-based approaches to perform micro chemical analysis of single neurons, studying neuropeptides and even metabolomics (Bai et al., 2011; Rubakhin et al., 2011; Yin et al., 2011; Ahmed et al., 2012; Cecala et al., 2012; Lanni et al., 2012; Nemes et al., 2012; Neupert et al., 2012; Tucker et al., 2012; Rubakhin et al., 2013). The group develops mass spectrometry protocols to measure peptides in individual neurons and individual neuronal processes, and they aspire to develop a low-concentration assay detecting peptides from subcellular microenvironments such as fractions of single cells.

Proteins from single cells can bind to antibodies coupled with fluorescence, and these antibodies may be spotted and arrayed on a chip in order to profile intra-cellular pathways (Shi et al., 2012). Thus, the foundations for different antibody arrays for single neuron specific pathways are set. A multidisciplinary, multimillion-pound Imperial College (UK) project, entitled “Single Cell Analysis” where David Klug is the Co-Director promotes research in this field. A study by Klug's group (Salehi-Reyhani et al., 2011) presented the development of an all-optical platform to analyze protein copy numbers in single cells, via microfluidic antibody capture detecting proteins using Total Internal Reflection Fluorescence (TIRF) microscopy. This methodology is particularly effective in studies of genetically labeled proteins or protein complexes in single cells. Willison and Klug advanced this to the use of the MAC chip; a “label free microfluidic antibody capture chip platform to quantify copy numbers of many proteins from a single cell in multiplexed single assay format” (Willison and Klug, 2013).

Another mind-blowing study describes the Nano needle biosensor that can be injected in living cells (Esfandyarpour et al., 2013a,b). The total diameter of the sensor is about 100 nm, and key element of this device is a 10 nm wide annular gap at the end of the needle. Any change in the population of molecules in this gap would result in a change of impedance across the gap, where single molecule detection should be possible. Flowing the solution containing the target molecules over arrays of such structures, where each one has its own integrated circuitry, “real-time” detection (for several minutes) of label free molecules can be attained without sacrificing sensitivity. The study concludes that the fully developed sensor would directly monitor protein quantities within a single living cell.

In conclusion, although the morphological diversity of neuronal cell types is a century old known secret, understanding the molecular complexity of terminally differentiated neurons has been achieved in the last years. The methodological advancements toward complete single neuron signature come from a series of science branches, unveiling the secrecy neuron by neuron. A transparent mouse brain or even a human brain slice, labeled with fluorescent mRNA or protein probes, or even stained for different neuronal circuits, plus the tools to isolate each one for single cell transcriptomics or proteomics studies, was in the realm of science fiction till last year. Now we can start dreaming big: dreaming of live transparent brain slices, where single neurons fluorescent for the mRNAs of choice could be analyzed for their proteome changes. We could design experiments with live transparent brain slices injected with Nano sensors that monitor proteome and transcriptome at real time. We could go further to work with CLARITY transparent Brainbow whole animal experiments (Livet et al., 2007; Chung and Deisseroth, 2013). We would then start to grasp the finer details regarding neurons; how they decide when and how to follow different developmental and environmental cues, how they pick the neurons they communicate with, what changes according to sex or hemisphere positioning, what changes happen in the diseased cells and how all these translate into cognition and behavior. Processing all these data of the asymmetrical-neuron studies will finally carve out the asymmetrical human brain. We might be lucky enough to see it realized in our times.
